# Finding the optical properties of plasmonic structures by image processing using a combination of convolutional neural networks and recurrent neural networks

**DOI:** 10.1038/s41378-019-0069-y

**Published:** 2019-06-17

**Authors:** Iman Sajedian, Jeonghyun Kim, Junsuk Rho

**Affiliations:** 1 Department of Mechanical Engineering, Pohang University of Science and Technology (POSTECH), Pohang, 37673 Republic of Korea; 2 Department of Chemical Engineering, Pohang University of Science and Technology (POSTECH), Pohang, 37673 Republic of Korea

**Keywords:** Nanophotonics and plasmonics, Nanophotonics and plasmonics

## Abstract

Image processing can be used to extract meaningful optical results from images. Here, from images of plasmonic structures, we combined convolutional neural networks with recurrent neural networks to extract the absorption spectra of structures. To provide the data required for the model, we performed 100,000 simulations with similar setups and random structures. In designing this deep network, we created a model that can predict the absorption response of any structure with a similar setup. We used convolutional neural networks to collect spatial information from the images, and then, we used that data and recurrent neural networks to teach the model to predict the relationship between the spatial information and the absorption spectrum. Our results show that this image processing method is accurate and can be used to replace time- and computationally-intensive numerical simulations. The trained model can predict the optical results in less than a second without the need for a strong computing system. This technique can be easily extended to cover different structures and extract any other optical properties.

## Plasmonic structures: predicting optical properties with neural networks

Neural networks can accurately predict the optical properties of plasmonic structures; engineered nanostructures with unique and interesting optical properties. Researchers from South Korea’s Pohang University of Science and Technology (POSTECH), led by Junsuk Rho, linked two machine learning algorithms to collectively extract spatial information from 2D images of structures and use this information to determine their light absorption characteristics. The team first trained their system on simulated data sets of structures with known absorption spectra, which the system then used to correctly deduce the optical properties of almost all 10,000 newly simulated structures with which it was provided. The neural networks processed each image in less than one second, without the need for powerful computers. The team says that by altering the training data, their system can adapt to work with other types of structures and optical properties.

## Introduction

Recently, the use of deep neural networks in solving scientific problems has increased, such as for finding exotic particles in high-energy physics^1^ and predicting the sequence specificities of DNA and RNA in biology^2^. In nanooptics, these networks have been used in the design and inverse design of nanophotonic structures^3–6^ and for the design of chiral metamaterials^7^.

Research studies regarding the optical properties of nanostructures all share a common theme, that is, connecting the geometric parameters of a structure to its optical response, for example, relating the radius and height of a cylinder to its reflection response. Although this subject is interesting, the structure designs are limited by the few geometric parameters.

Here, we show how these geometric limitations can be overcome by simply using a 2D image of the desired structure. We show that our model can predict the optical response of any given structural image in less than a second without the need for a strong computation system. We first demonstrate how 3D structures can be converted into 2D images, and then, we show how we use image processing to extract the optical response of the given structure. This method allows much more freedom in the design of a structure compared with using only a few geometric parameters.

The idea of using neural networks in image processing has proved to be efficient, appealing to a number of complex problems such as image classification^8^ and image labeling^9^. Image classification is performed by convolutional neural networks (CNN)^8^, which are advanced forms of neural networks designed to extract data such as lines, curves, edges, and structure orientation from images^10^. However, image labeling is performed by combining CNNs with recurrent neural networks (RNN)^11^, which are known for their ability to find relationships in data^12^.

**Fig. 1:**
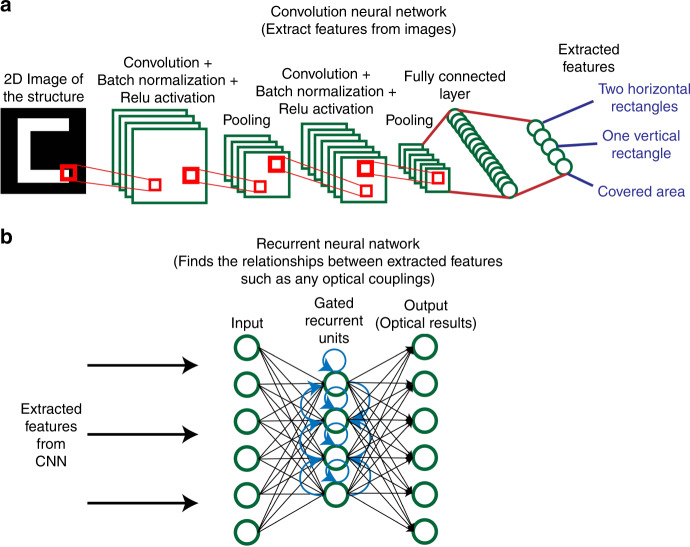
Structure of the deep learning model. **a** Convolutional neural networks are used to extract spatial features from an image of a structure by extracting data from smaller parts of the image. **b** Recurrent neural networks are used to find the required optical response based on the features extracted from the CNN

We can use these techniques to extract the optical response of a 3D structure from simple 2D images (Fig. 1). Here, the optical response that we chose is the absorption curve of a structure. Although CNNs are mostly used as a tool for classification, as absorption curves are numerical values, our problem is a linear regression problem^13^.

From previous research, we already know that the optical response of a structure is owing to its shape, size, and design. The CNN extracts the spatial information from the image, such as the shapes and their locations, and then, the RNN finds the relationship between the image and its optical features.

## Results

CNNs consist of layers that extract spatial information from images. These networks extract this information by analyzing subsections of the whole image and using the results as new input data for other layers (Fig. 1). CNNs usually consist of an input layer, convolutional layers, pooling layers, dropout layers, fully connected layers, and an output layer. Each layer can be switched on or off according to the problem at hand^13,14^. RNNs use different kinds of layers. Here, we used a gated recurrent unit (GRU) layer^15^, which was more efficient than using long short-term memory^16^.

### Input data

With the goal of extracting optical responses from an image, here, we focused on the absorption response, but this method can be extended to any result as required. To create a 2D image of a 3D structure, we used the fact that if a structure is uniform in one dimension, the structure can be defined in 2D space by its cross-section. For example, an infinite cylinder can be approximated by a 2D circle. In machine learning, values that are constant over all of the input data can be omitted. Thus, under the assumption that all the structures have the same thickness, this dimension can be omitted from the input data.

**Fig. 2:**
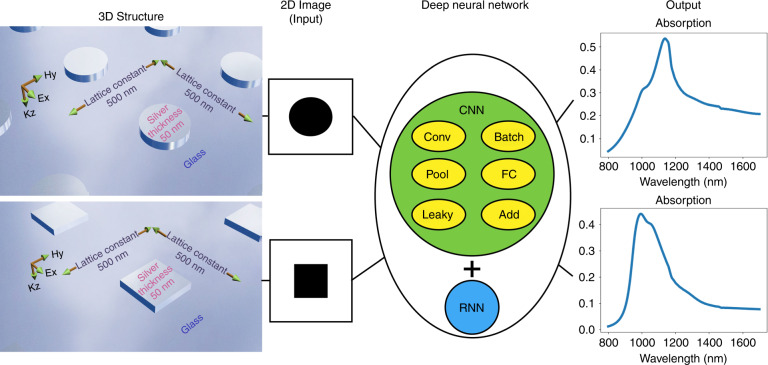
Schematic of the process, from the 3D structure to the absorption curve output. Image processing can predict the absorption curve from the 2D image of a given structure. A 3D structure with similar properties and different shapes can be modeled as 2D images. All the geometric properties such as the lattice constant, material type, polarization, and thickness should be the same. Under these conditions, the shape of the structure can be shown as a 2D image. This image is fed into a deep neural network. The network can predict the absorption curve from the given images. The deep neural network is a combination of a CNN and an RNN

Here, to create random structures, we fixed certain geometrical properties and allowed complete freedom of the shape. The structure was made of silver, the substrate was glass, the height of the structure was 50 nm, the lattice constant was set to 500 nm and the polarization of the source was fixed. Periodic boundary conditions were chosen to simulate an infinite array. A schematic of the process is shown in Fig. 2. By fixing these parameters, we did not change the physics of the problem, and all of the necessary geometric information was maintained. That we used only one material type allowed us to use black and white images for the input. If we wanted to use more material types, we should use color images, which leads to three additional channels for the input data (for red, green, and blue). Using black and white pictures means that wherever there is silver in our structure, there are black pixels in the input image, and wherever there is no silver, there are white pixels in the input image. The image resolution is 100 × 100 pixels, so the resolution is high enough to not lose any detail from the structure, but the amount of data are small enough to avoid memory problems when trying to compile the model.

The resolution of the images was not related to the meshing of the simulations. Accurate optical results were first simulated with a fine mesh, and then, the image of the structure was extracted at a lower resolution for image processing. Although this procedure causes a certain amount of data loss, a higher resolution image leads to a much heavier computation load with relatively little gain. Therefore, choosing the resolution is largely dependent on the computational power available.

**Fig. 3:**
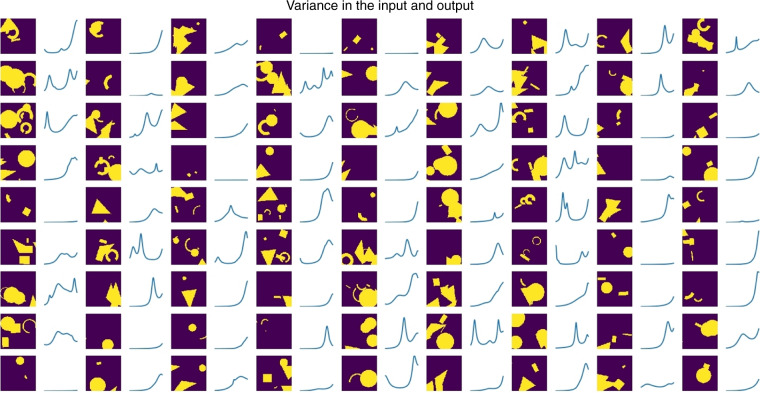
A selection of the images of random structures used as input to teach the model and their corresponding simulated absorption curves. The light areas represent the silver structure and dark represents empty space. The absorption curves are shown for wavelengths of 800 nm to 1700 nm. We removed the axes so that more structures can be seen

To ensure the accuracy of the model, using Lumerical, a commercial finite-difference time-domain (FDTD) simulation package, 100,000 simulations were prepared and subjected to the model. Certain structures and their corresponding absorption curves are shown in Fig. 3. Each simulation was run with 1000 frequency points, giving an output of 1000 nodes for the model.

The input data of both the geometric designs and the physical results had to include enough variance so that the model could predict unseen structures, so the input parameters were defined as follows and selected randomly for each shape:

- Number of shapes in each structure (was chosen randomly from one to six shapes)
- Type: circle, triangle, rectangle, ring, or polygon.
- Dimensions: width, radius, and length.
- Position: *x* and *y*.
- Rotation: 0–360 degrees.

This definition allowed enough freedom to cover all possible outcomes.

### Model layout and implementation

In preparing the model, there were a large number of factors to optimize. Here, we optimized:

- The number of convolution layers.
- The number of nodes in each convolution layer.
- The shape of the strides used in the convolution layers.
- The layout of the layers.
- The number of neurons in the fully connected layers.
- The activation functions.
- The loss function
- The optimizer and its hyperparameters.

We started by splitting our data into three parts. Sixty percent of the data was used to train the model; this is the training data set. Thirty percent of the data was used to test the model; this is the test data set. The final ten percent was used as the validation data set for validating the model. The names of the validation and test data sets are conventional, and certain researchers swap these names. To assess the efficiency of the model, we changed parameters such as the number of layers and checked the model on the test data set. The final model chosen was the model with the lowest loss on the test data set (loss is defined in equation 1). That model was then used with the validation data set. The validation stage assures that the model works on unseen data.

The loss function was defined as the mean squared error^17^:

1 $$M.S.E. = \frac{1}{n}\mathop {\sum}\limits_{i = 1}^n {\left( {Y_i - P_i} \right)^2}$$

In equation 1, *Y* is the vector of real values, and *P* is the vector of predictions. Batch normalization and weight regularization were used to avoid overfitting.

**Fig. 4:**
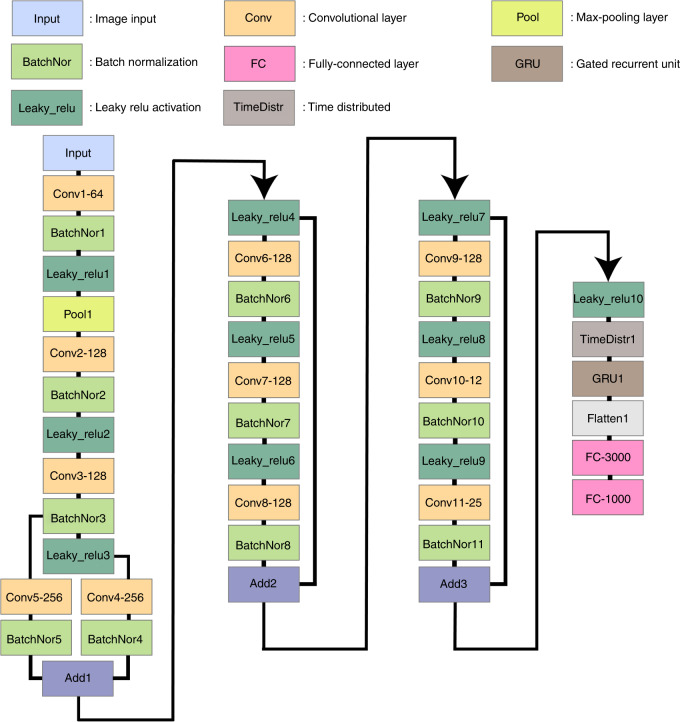
Deep learning model layout. The boxes show the filters used in each layer. A total of 500 epochs were run in the training stage, with a learning rate of 0.0001 and Nesterov Adam as an optimizer. This model is a combination of a ResNet CNN model and an RNN model. The output is from the final fully connected layer with 1000 nodes, where each node is a frequency point in the absorption curve

The final layout for the model is shown in Fig. 4. The model is a combination of a residual network CNN, known as ResNet^18^, and a small RNN. The residual network CNN is composed of groups of convolutional layers followed by batch normalization (for regularization) and a leaky relu layer for activation. The first group is followed by a pooling layer for additional regularization. We used shortcuts in three parts of our model. The shortcut is defined as adding the output of a layer to the output of the next layer. In this way, the gradients can flow back through this shortcut to earlier layers, and deeper networks can be designed. As an example in the middle part of Fig. 4, we added the output of leaky relu layer no. 4 (or the input of convolution layer no. 6) to the output of batch normalization layer no. 8. This technique was shown by other researchers^18^ to give a better performance than those of other model designs. After leaky relu layer no. 10, we used a time distributed layer to prepare the output of the CNN network for the input of the RNN network. This method is a coding technique, as RNNs require a special input shape. For the RNN network, we used a GRU layer and connected this layer to the fully connected layers (with 3000 nodes) with a flatten layer. A flatten layer is another coding technique for preparing the output of RNNs for the input of fully connected layers. The fully connected layer with 3000 nodes is then connected to the fully connected layer with 1000 nodes, which is the output layer. We used 1000 frequency points for our simulations. Thus, our network connects the input image (the input of convolution layer no. 1) to the output absorption curve (the output of the fully connected layer with 1000 nodes).

The ResNet architecture has a few key properties, specifically that a very deep network can be defined without getting a very small gradient^18^, and there are fewer memory problems owing to the lower number of trainable parameters compared with other models owing to a shortcut connection that results from the addition of a layer to one or more of the next layers.

Owing to the complexity of the problem, although various layouts for the model were tested, none of these layouts produced acceptable losses. Thus, to teach the model, more input data were needed. To prepare 100,000 simulations of input data, a CPU server with 28 Intel Xeon CPU E5-2697 v3 2.6 GHz cores was used, and the simulations took 15 days to run.

To implement the model, Keras with the Tensorflow backend was used, and the code was written in Python. A GPU server with two GTX 1080ti graphics cards was used for the implementation of the deep learning model, and the deep learning model took ~1 week to run. The same model would take much longer to run on a CPU server.

**Fig. 5:**
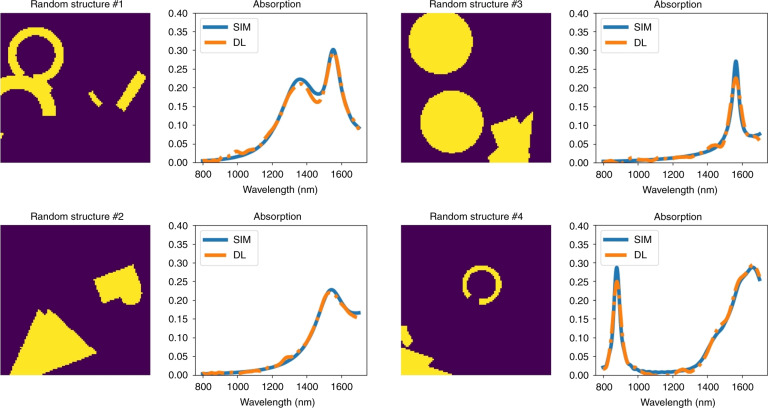
Results of applying the final deep learning model on a sample of random structures from the validation data set. The model predicted almost all 10,000 results from the validation set exactly. The solid blue lines show the absorption curves obtained from the simulation package, and the dotted orange lines show the absorption curves predicted by the deep learning model. These curves show a one to one comparison of predicted absorption value vs real absorption value for each frequency

**Fig. 6:**
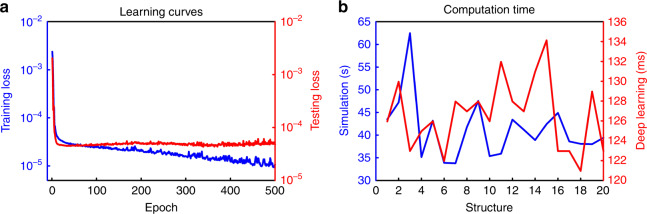
Neural network model training and computational efficiency. **a** Training loss and testing loss as the model trains. The code saves the model with the lowest loss on the test data set as the best model. Both of the *y* axis curves are shown on a logarithmic scale. **b** A comparison of computation time between simulation and deep learning on 20 different structures

**Fig. 7:**
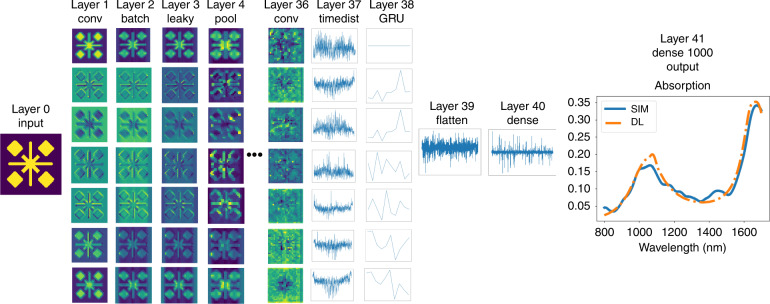
Output of a few selected layers of the deep learning model. This figure shows what happens inside the model. The first layer is the input image of the structure, and the final layer is the desired output physical quantity. The model consists of 42 layers, and each of these layers consists of a number of layers themselves. For better comparison, the final layer is combined with the expected results from simulation

The lowest loss achieved for the validation data set after 500 iterations was 4.2591 × 10^−05^. The results of certain structures are shown in Fig. 5. As the figure shows, the model correlates very well to the simulated absorption curves. The absorption curves shown in Fig. 5 are the predicted values of absorption for each frequency by deep learning compared with the computed values of absorption for each frequency by simulation. The prediction loss can be further reduced by fine-tuning the model hyperparameters or layer design. However, achieving such a goal may take a very long time, as each run of the model takes a long time. Thus, based on a personal evaluation of the results or based on the problem type, one can decide whether the final results are acceptable or can be improved. This procedure is the same as that in FDTD simulations, in which finer meshing leads to more accurate results in exchange for heavier computation load. It is not possible to increase the accuracy for a specific part, and any change to the model is applied to all the outputs. In Fig. 6a, we show the performance of the model on the train and test data sets as the model progresses. To show the steps inside each layer of the deep learning model and how the model extracts features from the image, the output of each layer is shown in Fig. 7 by simulating a specific design. As the model goes deeper, a higher level of features are extracted. For example, in layer 4, the CNN decided that the edges of squares have a more important effect on the outcome and thus neglected the inside of the squares. A more detailed analysis of feature extraction using CNNs can be found in the literature^19^.

The final model can predict the absorption curve of any given structure in less than a second, the same as any other machine learning model. The prediction of the results does not require much computational power, so the prediction can be easily performed at home on most consumer PCs, remotely over a website and even on a smartphone. A user interface is needed to prepare the images for the input of the model. A comparison between the computation times of the simulation and deep learning model is shown in Fig. 6b.

## Discussion

The method introduced here can be easily adapted to cover other structures and predict any optical outputs. For example, to consider other thicknesses, simulations for the desired thicknesses can be performed, and then, the deep learning model can be used on the new data. Next, both the images and the thicknesses are input. A method to solve this problem is to add this extra factor to the fully connected layers. Thus, after extracting the spatial information from the image with the convolution layers, the thickness parameter can be added by concatenating its array with the first fully connected layer. This method leads to a model that can predict the optical responses of different structures with different thicknesses. Different kinds of materials can also be used by using color input images, where each material can be assigned a specific color. To predict other optical responses, the results desired in the training stage can be input to the model. If we want the model to find any new relationships between the input and the output (such as the effect of thickness and using structures with different materials), we should provide the model with enough relevant data so that the model can learn from the data. Based on the complexity of the desired relationship, the amount of data can be small or large, which can be determined by trial and error in the training stage.

The model above can also be used as a discriminator for predicting structures with desired the absorption curves, basically performing the same process but in reverse, by using generative adversarial networks (GANs). GANs consist of two neural networks, one network that suggests a design, called the generator, and one network that verifies the design, called the discriminator. The generator starts from noisy images and improves with help from the discriminator. By using a good model for the discriminator, such as the model that we introduced here, structures can be predicted to fit the desired optical properties^20–22^. This model can be extended to completely replace an FDTD in a commercial package by providing enough data and training structures or even using 3D structures as the input of the model, which means using 3D convolutional layers and similar deep learning layers for 3D matrices. However, this extension means a huge computational demand that is currently impossible for normal users and maybe even large companies but may be possible in the near future.

## Conclusions

Here, a method using image processing instead of numerical simulations to determine the optical properties of structures was introduced. First, the technique for converting 3D structures into 2D images was discussed. These images were then used as inputs to the neural network model for image processing, and unseen data were used to check the accuracy of the model. We discussed how this model can be generalized for other structures and how the model can be used to predict structures with desired optical properties. The final model can predict the optical response of any structure from an image in less than a second without the need for extensive computing power.
